# Hydrogen sulfide-releasing hydrogels: a new perspective on clinical translation

**DOI:** 10.4103/mgr.MEDGASRES-D-25-00245

**Published:** 2026-09-12

**Authors:** Yue Xu, Zongchi Liu, Zongpu Xu, Yu Zhu

**Affiliations:** 1 Department of Neurosurgery, The First Affiliated Hospital, Zhejiang University School of Medicine, Hangzhou, Zhejiang Province, China; 2 Department of Neurosurgery, Sir Run Run Shaw Hospital, Zhejiang University School of Medicine, Hangzhou, Zhejiang Province, China; 3 Institute of Applied Bioresources, College of Animal Sciences, Zhejiang University, Hangzhou, Zhejiang Province, China; 4 Zhejiang Provincial Key Laboratory of Silk and Silk Protein New Materials, Zhejiang University, Hangzhou, Zhejiang Province, China

**Keywords:** angiogenesis, biomedical, cardiovascular, H_2_S, hydrogel, hydrogen sulfide, metabolism, neuroprotection, vasodilation, wound healing

## Abstract

Hydrogen sulfide (H_2_S), an endogenous gaseous signaling molecule, plays crucial roles in physiological regulation. However, efficient delivery and controlled release of H_2_S remain major challenges for clinical application. Hydrogels, with the unique three-dimensional network structure, provide a versatile platform for H_2_S loading and controlled release, offering a promising strategy to address these limitations. This article reviews the fabrication strategies of H_2_S-releasing hydrogels, including H_2_S donors, hydrogel properties, and incorporation strategies. In addition, biomedical applications of H_2_S-releasing hydrogels, including neurological therapy, cardiovascular therapy, regulation of vascular tone and angiogenesis, bone metabolism, wound healing, and others, are discussed. By integrating current advances, the article aims to provide new perspectives for the further development and clinical translation of H_2_S-releasing hydrogels.

## Introduction

Hydrogen sulfide (H_2_S) is a colorless, highly toxic, and acidic gas characterized by a distinctive rotten egg odor. Historically regarded as an environmental hazard over the centuries,^1^ advancing research during the last two decades has revealed its critical involvement in multiple pathophysiological processes within mammalian tissues.^2^,^3^ H_2_S functions as an endogenous modulator and is now recognized as the third gaseous signaling molecule,^1^ alongside carbon monoxide (CO) and nitric oxide (NO) (**Figure 1**).

**Figure 1:**
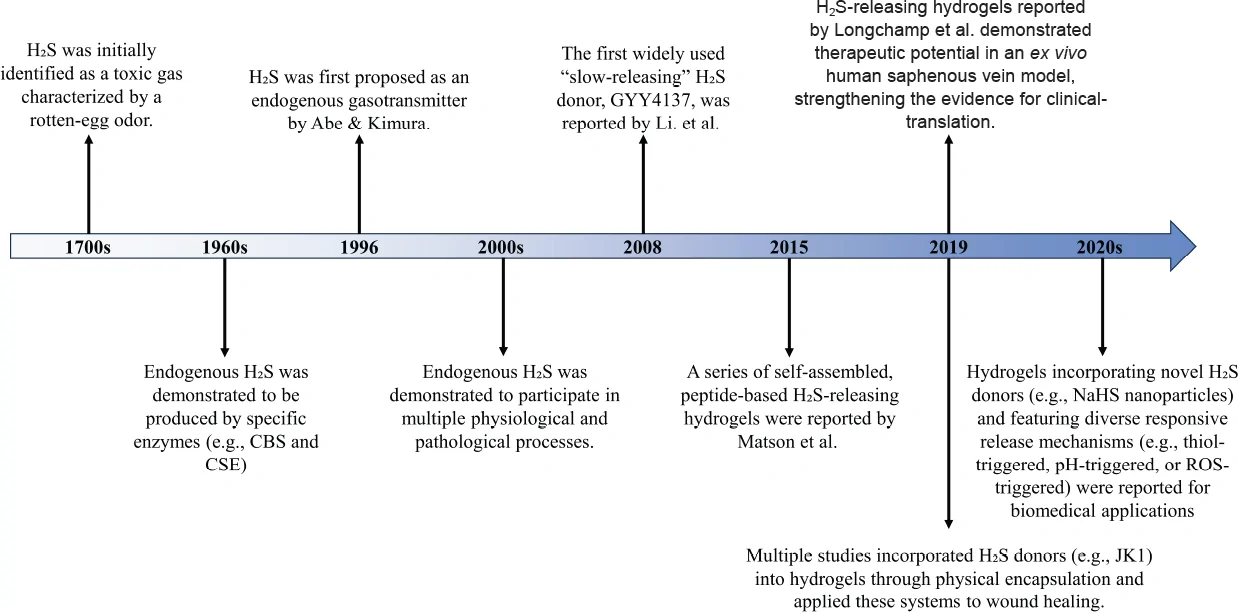
Milestones in H_2_S biology and H_2_S-releasing hydrogel development. In the 1700s, H_2_S was initially identified as a toxic gas. In the 1960s, enzymes like CBS, and CSE, were identified. In 1996, Abe and Kimura first proposed H_2_S as an endogenous gasotransmitter. In the 2000s, H_2_S biological effects were explored. In 2008, the slow-releasing H_2_S donor GYY4137 was reported, enabling controlled H_2_S delivery. From around 2015, self-assembled and peptide-based H_2_S-releasing hydrogels emerged, followed by the incorporation of various H_2_S donors into hydrogel matrices for biomedical applications. In 2019, *ex vivo* studies provided evidence supporting translational potential. In the 2020s, hydrogels integrating novel H_2_S donors and stimuli-responsive release strategies have been developed for expanded biomedical applications. Created with PowerPoint 16.101.3. CBS: Cystathionine β-synthase; CSE: cystathionine γ-lyase; H_2_S: hydrogen sulfide; NaHS: sodium hydrosulfide; ROS: reactive oxygen species.

The production of H_2_S can be categorized into environmental and endogenous sources. Environmental sources include volcanic eruptions, decomposition of crustal rocks, sulfate-reducing bacterial activity, oil and natural gas extraction, and emissions from sewage and waste.^4^,^5^ In mammalian cells, H_2_S is synthesized through specific enzymatic pathways under physiological conditions (**Figure 2**). The key enzymes responsible for endogenous H_2_S production include cystathionine γ-lyase (CSE), cystathionine β-synthase, and 3-mercaptopyruvate sulfurtransferase, which differ in tissue distribution and catalytic mechanisms.^6^ CSE is primarily distributed in the cardiovascular system and liver. Although low levels of CSE mRNA have been detected in the brain, H_2_S production there appears to be largely independent of CSE activity. In contrast, cystathionine β-synthase is the predominant H_2_S-producing enzyme in the brain and nervous system, catalyzing the condensation of serine and homocysteine to produce cystathionine. 3-Mercaptopyruvate sulfurtransferase is mainly localized in the mitochondria, participating in cysteine catabolism and cyanide detoxification.^7^,^8^ Additional sources of H_2_S in mammals include dietary intake, the gut microbiota, and non-enzymatic reactions.^7^ Compounds such as allicin from garlic and natural isothiocyanates from cruciferous vegetables can be ingested and generate H_2_S via thiol activation. The gut microbiota effectively metabolizes sulfate to generate H_2_S, constituting a major source of H_2_S in the intestine. Non-enzymatic pathways also contribute to H_2_S production in mammals; for instance, red blood cells can reduce elemental sulfur to H_2_S using reducing equivalents derived from glucose oxidation.^8^

**Figure 2:**
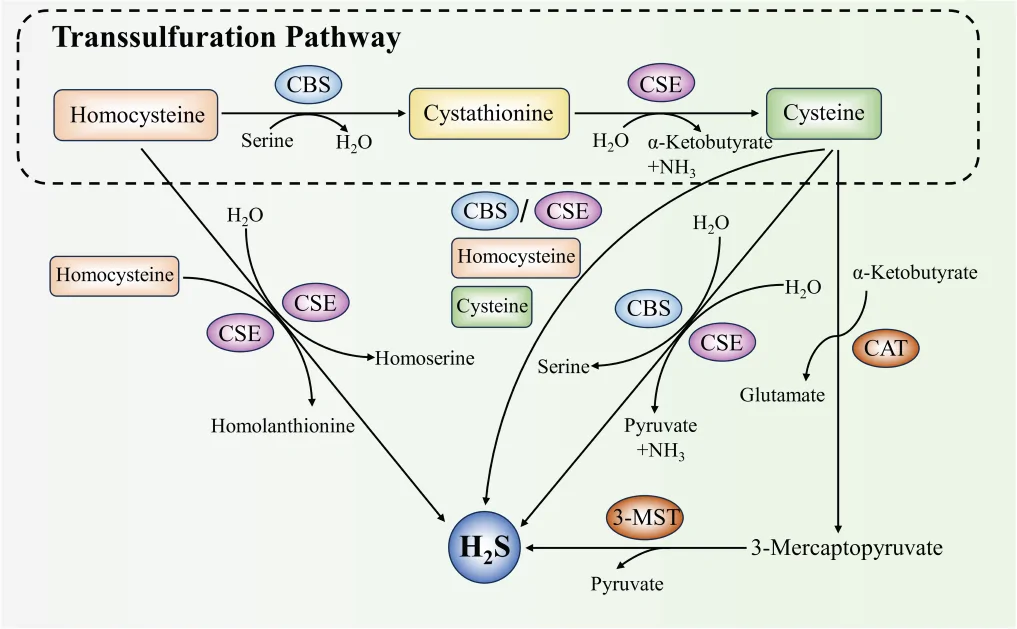
Enzymatic pathways for the biosynthesis of endogenous H_2_S. Cysteine and homocysteine serve as substrates for CSE and CBS, which enzymatically convert them to H_2_S. Cysteine is also catabolized via the CAT/3-MST pathway to generate H_2_S. Created with PowerPoint 16.101.3. 3-MST: 3-Mercaptopyruvate sulfurtransferase; CAT: cysteine aminotransferase; CBS: cystathionine β-synthase; CSE: cystathionine γ-lyase; H_2_S: hydrogen sulfide.

Endogenous H_2_S has been demonstrated to play essential roles in multiple mammalian systems, particularly the cardiovascular and nervous system, and in inflammatory and immune response^6^,^9^,^10^ (**Figure 3**). Among its physiological functions, H_2_S exerts significant regulatory effects on vasodilation. Earlier studies suggested that H_2_S activates adenosine triphosphate-sensitive potassium channels in vascular smooth muscle cells, thereby promoting vasodilation and reducing peripheral resistance.^11^,^12^ Several studies have indicated that H_2_S may also be synthesized by endothelial cells and function as an endothelium-dependent hyperpolarizing factor in specific vascular beds.^13^,^14^,^15^ In addition, H_2_S exerts a neuroprotective role by modulating glutamatergic and γ-aminobutyric acidergic neurotransmission, inhibiting microglial activation, and reducing oxidative stress. However, under pathological conditions, dysregulation of H_2_S metabolism is associated with multiple disorders, such as hypertension, heart failure, Alzheimer’s and Parkinson’s diseases, renal dysfunction, hepatic fibrosis, inflammation, immune dysregulation, and cancer progression.^16^,^17^ Low levels of H_2_S are recognized hallmarks of specific diseases and conditions, suggesting that H_2_S may hold therapeutic potential in their treatment.^18^ Given short half-life, high reactivity, and low stability of H_2_S in biological environments, the development of delivery systems has emerged as a critical focus in research and clinical applications. Recently, various H_2_S-releasing delivery platforms have been developed and applied in research, including H_2_S-releasing polymers, microparticles and fibers, nanocomposite scaffolds, etc.^19^,^20^ Among them, hydrogels attract considerable attention due to their wide applicability and tunable properties.

**Figure 3:**
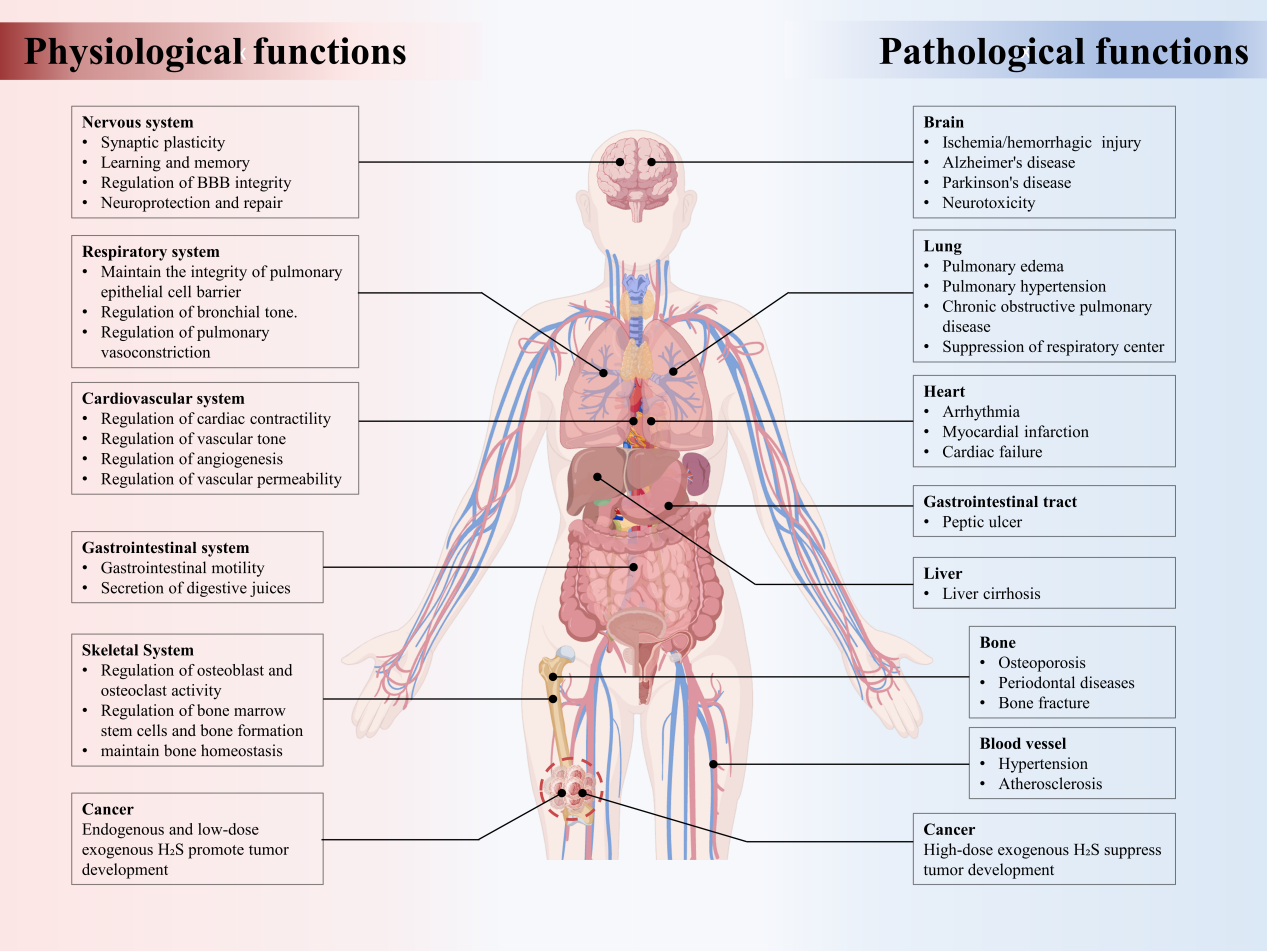
Physiological and pathological roles of H_2_S in major organ systems. Endogenous H_2_S exerts diverse physiological functions, including modulation of synaptic plasticity and neuroprotection in the nervous system, maintenance of pulmonary epithelial integrity and bronchial tone, regulation of cardiac contractility, vascular tone, angiogenesis, gastrointestinal motility, and bone homeostasis. Under pathological conditions or dysregulated concentrations, H_2_S is implicated in multiple diseases, such as cerebral ischemia and neurodegeneration, pulmonary edema, myocardial infarction and heart failure, gastrointestinal and liver disorders, osteoporosis, hypertension, and cancer. H_2_S also exhibits concentration-dependent dual effects in tumor biology. Created with PowerPoint 16.101.3 and BioGDP.com. BBB: Blood–brain barrier; H_2_S: hydrogen sulfide.

Hydrogels are materials formed by crosslinking hydrophilic polymers, consisting of a distinct three-dimensional network. This structure imparts several advantageous properties to hydrogels, including high water absorption, excellent biocompatibility, appropriate mechanical strength, and sensitivity to environmental stimuli.^21^,^22^ Hydrogels can be classified as natural or synthetic based on their origin. Natural hydrogels are derived from naturally occurring polymers, including sodium alginate, hyaluronic acid (HA), chitosan, etc. These materials typically exhibit excellent biocompatibility and biodegradability, but generally possess weak mechanical strength.^23^ In contrast, synthetic hydrogels are fabricated from chemically synthesized hydrophilic polymers via chemical crosslinking, such as polyethylene glycol, polyacrylamide, polycaprolactone, and polyvinyl alcohol, which typically exhibiting enhanced mechanical properties.^23^ Synthetic hydrogels allow for precise performance tuning through molecular design and synthesis, enabling broader application potential. In recent years, hydrogels have exhibited significant potential in biomedical applications due to their diverse biological and structural properties. Their excellent biocompatibility, high water content, and tunable physicochemical properties make them ideal for replicating the extracellular matrix microenvironment.^24^ In tissue engineering, hydrogels function as scaffolds for cell culture and tissue regeneration, providing physical support and biochemical signals that promote cell proliferation, and differentiation.^25^ Furthermore, hydrogels also play roles in a wide range of biomedical applications, such as wound dressings, biosensors, and disease models.^26^

By incorporating H_2_S donors into hydrogel matrices, H_2_S-releasing hydrogels have been developed to achieve controlled, sustained release of H_2_S. These systems attract increasing attention and are widely investigated for their therapeutic potential in biomedical applications, including neuroprotection, cardiovascular therapy, protection against ischemia-reperfusion injury, wound healing, metabolic balance, tumor therapy and regulation of inflammatory responses. This review focuses on the fabrication strategies and biomedical applications of H_2_S-releasing hydrogels (**Figure 4**).

**Figure 4:**
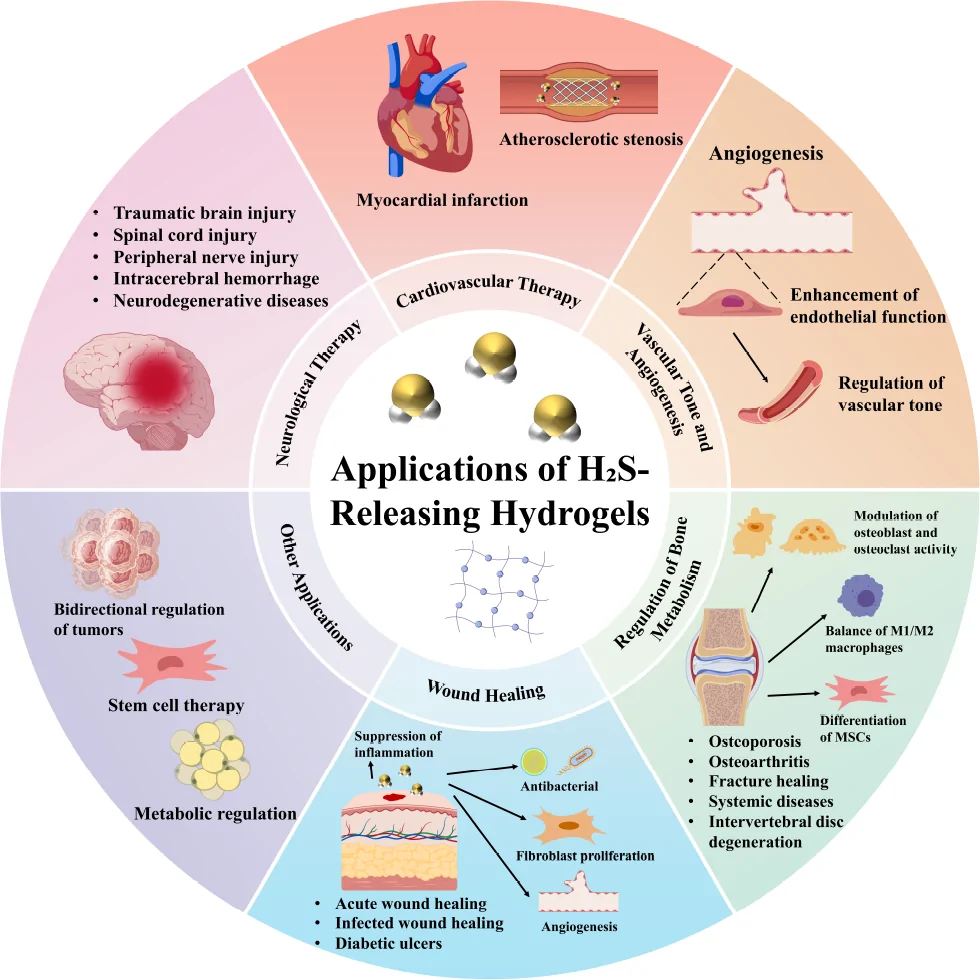
Biomedical applications of H_2_S-releasing hydrogels. H_2_S-releasing hydrogels exhibit broad biomedical applications in multiple systems, including neuroprotection, cardiovascular therapy, vascular tone regulation, angiogenesis, bone metabolism, wound healing, tumor regulation, metabolic modulation, and stem cell therapy. Created with PowerPoint 16.101.3 and BioGDP.com. H_2_S: Hydrogen sulfide; MSC: mesenchymal stem cell.

## Retrieval Strategy

A comprehensive literature search was performed via PubMed and Web of Science to identify studies related to H_2_S-releasing hydrogels and their biomedical applications. The search covered all publications from database inception to September 2025. The search was conducted using the terms “hydrogen sulfide,” “H_2_S,” “H_2_S donor,” “hydrogel,” and “therapy.” Additional related terms such as “neuroprotection,” “cardiovascular,” “metabolism,” “wound healing,” “antibacterial,” and “clinical translation” were used to broaden the search. Reference lists of the included articles and relevant reviews were also manually screened to identify additional studies. The inclusion criteria were as follows: (1) Investigations of H_2_S donors, H_2_S-releasing hydrogels, or hydrogel-based H_2_S delivery systems, (2) *in vitro*, *in vivo*, or *ex vivo* studies relevant to biomedical or therapeutic contexts, and (3) publications as original research articles, reviews, or systematic analyses. The exclusion criteria were as follows: (1) Studies lacking sufficient methodological details, and (2) studies not published in English or unavailable in full text.

## Strategies for Fabricating Hydrogen Sulfide-Releasing Hydrogels

### H_2_S donors

The gaseous nature and rapid diffusion of H_2_S present significant challenges for its targeted and controlled delivery.^27^ The development of H_2_S-releasing systems has attracted considerable attention, with numerous studies focusing on the design of effective H_2_S donors (**Figure 5**). H_2_S donors are classified into natural donors and synthetic donors according to the sources. Natural donors are primarily derived from Allium plants, such as garlic and onion. Currently, allicin from garlic has been widely applied in antibacterial, wound healing, anti-tumor and cardiovascular therapies.^28^,^29^ Diallyl trisulfide, a major active component of allicin, is generated through enzymatic hydrolysis following tissue disruption in fresh garlic. In the presence of thiols, diallyl trisulfide is reduced to allyl disulfide, which is subsequently further reduced to release H_2_S.^30^ Additionally, natural isothiocyanates in cruciferous vegetables and organic sulfides in mushrooms serve as natural sources of H_2_S.^31^,^32^

**Figure 5:**
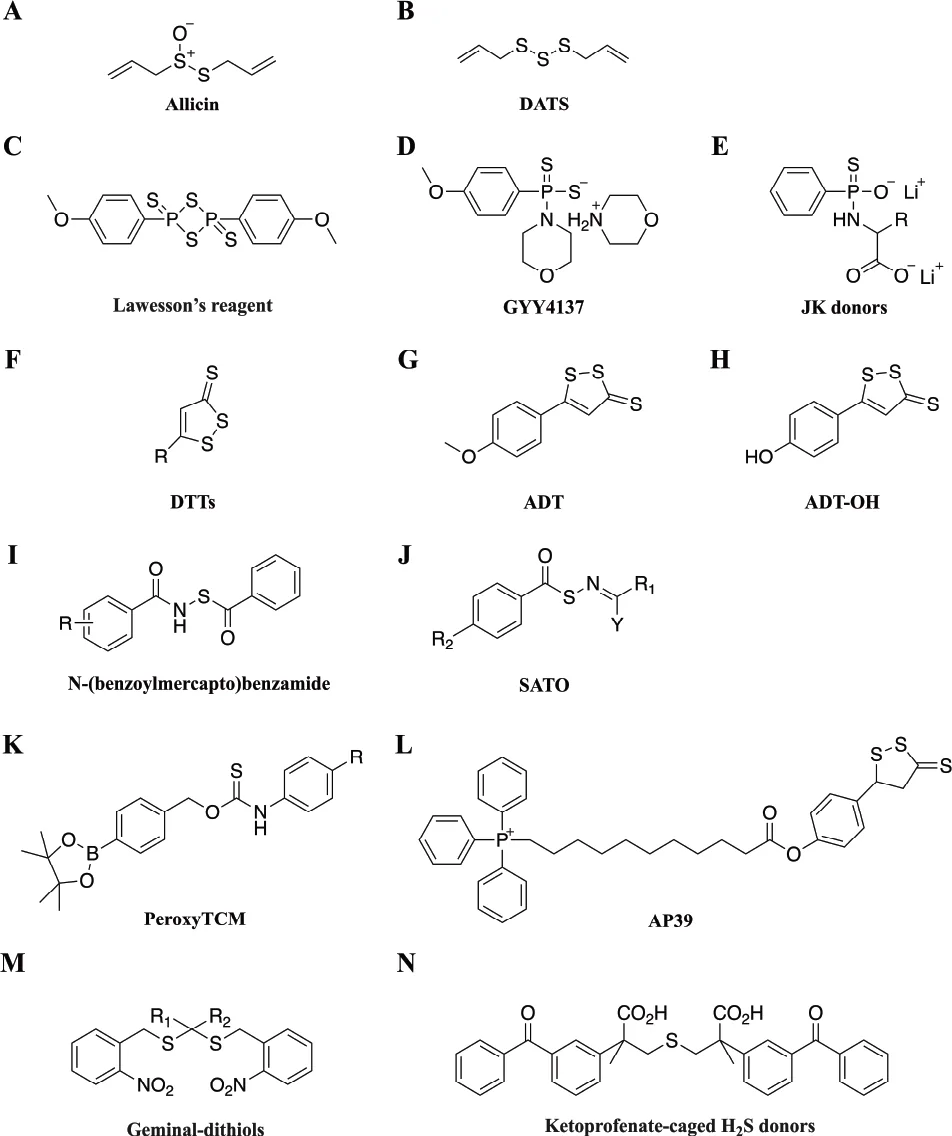
Common H_2_S-donating compounds, including natural and synthetic donors. Created with ChemDraw 20.0.038. ADT: Anethole dithiolethione; ADT-OH: 5-(4-hydroxyphenyl)-3H-1,2-dithiole-3-thione; DATS: diallyl trisulfide; DTTs: 3H-1,2-dithiol-3-thione derivatives; H_2_S: hydrogen sulfide; PeroxyTCM: peroxythiocarbamate; SATO: S-arylactylthioximes.

Inorganic sulfides, such as sodium hydrosulfide (NaHS) and sodium sulfide, are the earliest reported synthetic H_2_S donors.^33^ These compounds release H_2_S either directly upon dissolution in aqueous environments or through reactions with endogenous acidic species.^34^ However, their rapid release kinetics hinder sustained and controllable delivery, and their volatility and susceptibility to oxidation further limit their practical application.

To address these limitations, organic H_2_S donors are increasingly utilized in biological research. Currently, many activatable donors that release H_2_S in response to specific exogenous or biocompatible stimuli have been reported, including hydrolysis-activated donors, pH-controlled donors, thiol-activated donors and reactive oxygen species (ROS)-activated donors.^35^,^36^ Lawesson’s reagent and its derivatives, together with 3H-1,2-dithiol-3-thione derivatives, are classified as hydrolysis-activated donors. One notable example is GYY4137, a water-soluble donor synthesized via a straightforward reaction between Lawesson’s reagent and morpholine.^36^,^37^ GYY4137 was specifically designed to mimic the slow and sustained endogenous production of H_2_S under physiological conditions.^38^ It offers several advantages, including high chemical purity, low susceptibility to contamination, and a prolonged release profile, making it widely adopted in both *in vitro* and *in vivo* studies. Notably, Li et al.^39^ discovered that H_2_S release from GYY4137 is pH-dependent, with higher release at low pH conditions (pH 3.0). Based on this property, a series of pH-controlled GYY4137 derivatives (e.g., JK donors) were synthesized. JK donors exhibited pH-dependent H_2_S release, with a faster and greater H_2_S release under acidic conditions, while the release was slower and less pronounced at neutral or basic conditions.^38^ In one example, Yang et al.^40^ applied the JK-1 donor to aspirin-induced gastric mucosal lesions, demonstrating that acidic pH-triggered H_2_S release attenuated inflammation and oxidative stress, thereby preserving gastric mucosal function. The 3H-1,2-dithiol-3-thione derivative analog anethole dithiolethione is commonly used for the derivatization of H_2_S donors from existing drug molecules. For instance, esterification of diclofenac with 5-(4-hydroxyphenyl)-3H-1,2-dithiole-3-thione produced S-diclofenac, enhancing anti-inflammatory efficacy and mitigating gastrointestinal side effects.^41^

Thiol-activated H_2_S donors are compounds that release H_2_S upon activation by biological thiols, such as cysteine and reduced glutathione, with mechanisms involving thiol–disulfide exchange or thioester hydrolysis reactions.^35^ Common aryl thioamides and aryl isothiocyanates are typical representatives, exhibiting controlled H_2_S release capability.^36^ Several studies have also shown that the S–N bond in N-mercapto compounds is readily attacked and cleaved by thiols, suggesting their potential as H_2_S donors.^42^,^43^,^44^,^45^,^46^ To control the H_2_S release rate, Zhao et al.^42^ synthesized a stable H_2_S donor, N-(benzoylmercapto)benzamide, by protecting the thiol group. In addition, S-arylacylthioxoximes (SATOs), developed by Foster’s team,^43^ are a class of thiol-activated donors formed by the reaction of S-arylacylthiohydroxylamine with aldehydes or ketones, releasing H_2_S in the presence of cysteine. The functions of SATO donors for clinical treatments, such as antibacterial activity, wound healing and regulation of endothelial function, have been widely reported.^44^,^45^,^46^

ROS-activated H_2_S donors are a class of compounds capable of specifically releasing H_2_S in oxidative stress environments. These donors respond to elevated intracellular ROS levels, enabling precise H_2_S delivery and exerting antioxidant, anti-inflammatory, and cytoprotective effects in pathological conditions. Peroxythiocarbamate (PeroxyTCM), developed by Zhao and Pluth,^47^ is a ROS-activated donor based on thiocarbamates. Its design involves ROS-triggered release of carbonyl sulfide, which is then converted to H_2_S by carbonic anhydrase. These donors are stable in aqueous solution and selectively release H_2_S in the presence of ROS, superoxide, or peroxynitrite, providing significant protection against oxidative damage in cellular models. Recently, PeroxyTCM has been incorporated into hydrogels for neural injury repair, where it dynamically modulates oxidative stress and inflammatory responses in the microenvironment, thereby promoting neural regeneration.^48^,^49^

Furthermore, photo-activated H_2_S donors and mitochondria-targeted donors have been developed in recent years. Photo-activated H_2_S donors are compounds that release H_2_S upon irradiation with specific wavelengths of light, enabling spatiotemporal control of H_2_S release for potential clinical applications. Common photo-activated donors include gem-dithiols, ketoprofen derivatives, and oxazanthone-based compounds.^35^ Meanwhile, the development of mitochondria-targeted H_2_S donors has provided new perspectives on the subcellular delivery of H_2_S. AP39, which is targeted to the mitochondria by triphenylphosphonium, is the most well-established targeted H_2_S donor.^50^ Szczesny et al.^50^ demonstrated that AP39 exerted concentration-dependent effects on mitochondrial activity: at lower concentrations (30–100 nM), it enhanced mitochondrial electron transport and cellular bioenergetics, whereas at higher concentrations (300 nM), it exerted inhibitory effects. An appropriate concentration of AP39 can reduce intracellular oxidative stress, regulate the electron transport chain, and protect mitochondrial structure.^50^

### Properties and modification of hydrogels

The properties of hydrogels are intrinsically related to their three-dimensional network structures formed by the crosslinking of hydrophilic polymer chains (**Figure 6**). Crosslinking methods are generally classified into two main categories: chemical and physical. Chemical crosslinking involves the formation of stable covalent bonds, including free radical polymerization, click chemistry reactions, and enzyme-mediated crosslinking. In contrast, physical crosslinking relies on non-covalent interactions such as hydrogen bonding, ionic interactions, hydrophobic interactions, and π–π stacking. Crosslinking strategies profoundly influence the mechanical properties of hydrogels.^23^ Chemically crosslinked hydrogels exhibit high structural stability and mechanical strength but lack facile degradability, while physically crosslinked hydrogels offer enhanced biocompatibility but typically lower mechanical strength. The mechanical properties of hydrogels can be modulated by adjusting the crosslinking density, network architecture, and incorporation of additives. One study reported a trehalose-modified polyacrylamide hydrogel, in which network defects were repaired via covalent-like hydrogen bond interaction.^51^ This strategy increased the strength and stretchability from 19 kPa and 1150% to 81 kPa and 4100% and the fracture toughness from 474 J/m^2^ to 5116 J/m^2^, while also markedly enhancing tolerance to low temperatures and dehydration.^51^ Double-network hydrogels, constructed from a stiff and brittle first network combined with a soft and ductile second network, also represent an effective strategy to endow hydrogels with both high strength and toughness, thereby significantly improving their mechanical performance.^52^

**Figure 6:**
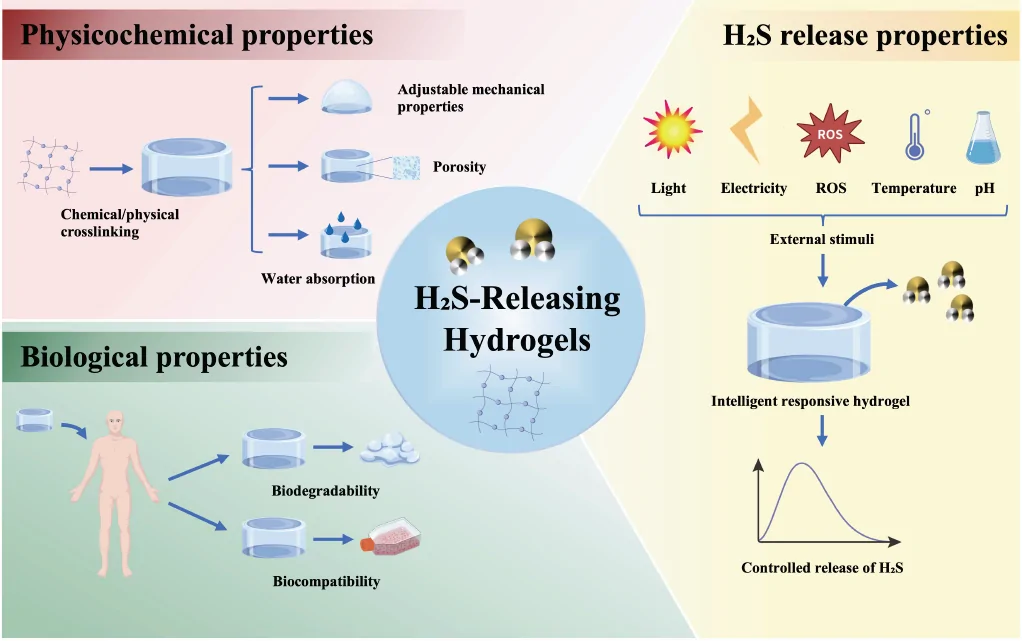
Properties of H_2_S-releasing hydrogels. H_2_S-releasing hydrogels exhibit tunable physicochemical properties determined by chemical or physical crosslinking, including adjustable mechanical strength, porosity, and water absorption capacity. Biologically, these materials offer biocompatibility and biodegradability for in vivo applications. Incorporation of H_2_S donors imparts stimulus-responsive release behaviors, whereby external triggers such as light, electricity, ROS, temperature, and pH activate intelligent hydrogel matrices to achieve controlled H_2_S delivery. Created with PowerPoint 16.101.3 and BioGDP.com. H_2_S: Hydrogen sulfide; ROS: reactive oxygen species.

The ability of hydrogels to absorb and retain substantial amounts of water without dissolving grants them a unique combination of fluidity and mechanical strength.^26^ This property originates from their three-dimensional polymer network, composed of chains rich in polar functional groups, including hydroxyl, carboxyl, amino, and amide groups. The hydrophilicity enables hydrophobic donor molecules to be incorporated into hydrogels through covalent bonding or non-covalent complexation, thereby enhancing the bioavailability.^20^

The microstructure and porosity of hydrogels are key determinants of their functional performance, particularly regarding pore size and porosity.^26^,^53^ Pore size directly affects molecular diffusion: hydrogels with small pores restrict macromolecular transport, making them suitable for controlled drug delivery, while macroporous hydrogels permit cell migration but compromise mechanical stability. Porosity, the percentage of void space in a material, is also critical.^54^ Enhancing the porosity of hydrogels facilitates the efficient diffusion of nutrients and oxygen, establishing a favorable microenvironment for cell proliferation and tissue regeneration.^53^ For example, Siboro et al.^55^ precisely regulated the “porous–dense” architecture of alginate hydrogels through crosslinker functional group design. Porous hydrogels exhibited more stable and sustained drug release profiles in comparison to dense hydrogels.

Hydrogels can also be modified to respond to changes in external stimuli, thereby altering physical structures or chemical properties to achieve intelligent drug release. Poly(N-isopropylacrylamide) (PNIPAM) is one of the most widely investigated thermo-responsive hydrogels, characterized by a lower critical solution temperature of approximately 32°C. PNIPAM undergoes an *in situ* sol–gel transition upon heating above this temperature, making it suitable for controlled drug delivery.^56^ However, PNIPAM inherently exhibits relatively low tensile strength, a limitation that can be markedly improved through the incorporation of nanoparticles, which simultaneously enhances its stimulus responsiveness.^57^ In particular, PNIPAM hydrogels loaded with gold nanoparticles demonstrate rapid heating under near-infrared irradiation, thereby synergistically facilitating controlled drug release.^58^

### Hydrogen sulfide incorporation strategies

Recently, increasing attention has been devoted to the application of hydrogels for H_2_S delivery. Two principal strategies have been established for incorporating H_2_S into hydrogels: physical encapsulation and covalent linkage.^18^ Physical encapsulation of H_2_S donors within the hydrogel matrix is the most convenient approach, in which H_2_S donors are directly dissolved or dispersed in the hydrogel precursor solution and subsequently entrapped within the crosslinked hydrogel network. In this case, the donors are retained mainly through weak interactions such as hydrogen bonding.^59^ Physical encapsulation offers advantages of operational simplicity and mild preparation conditions, minimizing disruption of both the H_2_S donor and the hydrogel matrix. The JK1 compound is frequently utilized as an H_2_S donor for physical encapsulation in current research and hydrogels incorporating JK1 exhibit a pH-dependent prolonged H_2_S releasing profile compared with free small-molecule donors.^60^,^61^,^62^ In addition, several studies reported the incorporation of a bio-macromolecular H_2_S donor, formed by conjugating small-molecule donors (e.g., 4-aminobenzothioamide and thiobenzoic acid) with keratin, into hydrogels for therapeutic applications.^63^,^64^

However, physically encapsulated H_2_S donors are prone to leakage or burst release upon hydrogel swelling, limiting the ability to achieve long-term and sustained H_2_S delivery. Covalently bonding H_2_S donors to hydrogel polymer chains provides a strategy to address this limitation. For example, several studies have developed a series of H_2_S-releasing hydrogels based on self-assembling peptides functionalized on the N-terminus with SATO donors.^65^,^66^,^67^,^68^ Compared with the peptide in solution, these hydrogels exhibited slower H_2_S release and minimal cytotoxicity, demonstrating promising potential for clinical applications, including anti-intimal hyperplasia, antimicrobial and osteoporosis treatment.^45^,^46^ Xiao et al.^69^ developed a light and amine dual-responsive thiobenzaldehyde precursor. Upon light activation, the precursor generated thiobenzaldehyde, which subsequently reacted with amines to form imines and release H_2_S. The thiobenzaldehyde precursor was then copolymerized with poly(ethylene glycol) methyl ether methacrylate and ethylene glycol dimethacrylate to construct a light-responsive H_2_S-releasing hydrogel, which exhibited slow H_2_S release under combined light and amine regulation and demonstrated good cytocompatibility. Liang et al.^70^ also developed a hydrogel capable of controlled H_2_S release by grafting 2-aminopyridine-5-thiocarboxamide onto partially oxidized alginate. H_2_S-releasing hydrogels fabricated through covalent linkage provide the advantages of stable donor incorporation and well-controlled release. Nevertheless, the associated chemical reactions are often complex and demand stringent control to ensure that both the structural integrity of the hydrogel and the desired H_2_S release profile are achieved.

Hydrogels incorporating H_2_S donors encapsulated within nanoparticles have also been reported. Dai’s team^49^,^71^ encapsulated PeroxyTCM within the ROS-responsive polymer poly(ethylene glycol)-poly(L-methionine) to form nanoparticles, which were subsequently loaded into hydrogels and successfully applied in the treatment of peripheral nerve injury and burn wounds. Fang et al.^72^ constructed an H_2_S-sustained release hydrogel by incorporating NaHS-encapsulated bovine serum albumin nanoparticles, which effectively promoted periodontal bone healing in diabetes. The above studies illustrated that combining nanoparticles with hydrogels enabled controlled H_2_S release by harnessing the sustained-release characteristics of nanoparticles together with the protective effects of hydrogels. Furthermore, the integration of nanoparticles imparted additional functionalities to hydrogels, such as stimulus responsiveness.

## Applications of Hydrogen Sulfide-Releasing Hydrogels

### Neurological therapy

Sulfides in the mammalian brain were first identified in 1989 by Warenycia et al.^73^ who reported a strong correlation between brain sulfide levels and mortality in rats exposed to inhaled H_2_S or administered NaHS. Notably, sulfides were also detected in the brains of untreated animals, prompting further investigation into the role of H_2_S in the central nervous system. Current evidence indicates that H_2_S performs diverse functions in the nervous system, including neuroprotection, regulation of synaptic plasticity, suppression of neuroinflammation, redox regulation, control of cerebrovascular function, and preservation of blood–brain barrier integrity.^7^,^74^,^75^ These properties present promising therapeutic targets for neurodegenerative diseases (e.g., Alzheimer’s disease, Parkinson’s disease), cerebral ischemia/hemorrhagic injury, acute neurological injuries, and disorders of the peripheral nervous system.^74^,^76^

Current research on H_2_S-releasing hydrogels mainly targets acute neurological injuries, including traumatic brain injury (TBI), spinal cord injury (SCI), peripheral nerve injury and intracerebral hemorrhage, with a focus on suppressing inflammation and promoting tissue repair. Neuroinflammation, pyroptosis, neurotransmitter imbalance, and oxidative stress are major drivers of secondary brain injury after TBI.^77^,^78^ Targeting neuroinflammation and pyroptosis has thus emerged as a promising therapeutic approach for TBI.^79^ A silk fibroin-based hydrogel encapsulating H_2_S (H_2_S@SF hydrogel) was developed to enable sustained H_2_S release and restore endogenous H_2_S homeostasis in injured neurons.^80^ The study showed the H_2_S@SF hydrogel significantly reduced the expression of proteins associated with pyroptosis and necroptosis in neurons after TBI in mice, thereby mitigating cerebral edema and neurodegeneration during the acute phase of injury. Behavioral assessments further confirmed that H_2_S@SF hydrogel treatment enhanced long-term neurological recovery, minimized tissue loss, and attenuated TBI-induced neuroinflammation.

Following SCI, the inflammatory microenvironment represents a major obstacle to neuronal regeneration and functional recovery.^81^ Studies indicate that H_2_S-releasing hydrogel treatment reduces oxidative stress and inflammation after SCI, protects neuronal cells, induces macrophage polarization toward the M2 phenotype, and promotes angiogenesis, thereby accelerating neural regeneration and functional recovery.^48^,^82^,^83^,^84^ Several studies combined H_2_S-releasing hydrogels with stem cell therapy, showing promising potential for neural repair and regeneration and suggesting that this approach may be viable for cell therapies.^82^,^84^ For instance, Albashari et al.^82^ developed a thermosensitive H_2_S-releasing hydrogel (PF-OMSF@JK) encapsulating dental pulp stem cells. Dental pulp stem cells encapsulated in PF-OMSF@JK displayed typical neuronal morphology and maintained high viability at the SCI site. In a different study, Zhao et al.^84^ developed a three-dimensional-printed gelatin methacryloyl scaffold loaded with H_2_S-preconditioned MSC-derived EVs. The scaffold markedly improved motor function recovery and axonal regeneration while reducing inflammation in SCI rats, offering a novel and effective strategy for promoting neuronal regeneration after SCI. Similarly, H_2_S donors have demonstrated comparable effects in modulating the neural regeneration microenvironment after peripheral nerve injury.^85^,^86^ One study developed a ROS-responsive hydrogel scaffold incorporating an H_2_S donor (PeroxyTCM). This system suppressed inflammation and oxidative stress, promoted angiogenesis, and restored mitochondrial function, thereby dynamically modulating the regenerative microenvironment and accelerating axonal growth and functional recovery.^49^

Hematoma compression and inflammatory responses after intracerebral hemorrhage can cause severe brain injury. H_2_S donors have been identified as potential therapeutic agents for brain injury following intracerebral hemorrhage.^87^,^88^ Zhang et al.^89^ reported that an injectable H_2_S-releasing silk fibroin hydrogel (H_2_S@SF) enables sustained H_2_S release and enhances endogenous H_2_S production, thereby reducing neuronal pyroptosis, alleviating cerebral edema and hemorrhagic injury, and improving neurological function after severe intracerebral hemorrhage. The H_2_S-releasing hydrogel represents a promising biomaterial with potential clinical applications in the treatment of intracerebral hemorrhage.

In addition, H_2_S donors exert distinctive molecular regulatory effects in neurodegenerative diseases.^74^,^90^,^91^ Studies have shown that both H_2_S levels and cystathionine β-synthase activity are markedly reduced in the brains of patients with Alzheimer’s disease, suggesting a potential association between H_2_S deficiency and cognitive and memory impairments.^90^ H_2_S donors have been reported to modify glycogen synthase kinase 3β via sulfhydration, thereby preventing abnormal tau protein phosphorylation and improving cognitive function in Alzheimer’s disease mouse models.^92^ Moreover, H_2_S donors significantly ameliorate learning and memory deficits and reduce β-amyloid plaque burden in the cortex and hippocampus.^93^ Other studies demonstrated that GYY4137 administration also increased synaptic protein levels and enhanced synaptic plasticity in Alzheimer’s disease mice.^94^,^95^ Similarly, H_2_S donors have been shown to mitigate dopaminergic neuronal loss in Parkinson’s disease. Mechanistically, this neuroprotective effect is mediated through reducing α-synuclein aggregation, alleviating oxidative stress and inflammation, inhibiting apoptosis, and promoting neuronal regeneration.^90^,^96^,^97^ The neuroprotective role of H_2_S has been confirmed in multiple Parkinson’s disease models, both *in vitro* and *in vivo*.^98^,^99^,^100^,^101^,^102^ However, research on the direct application of H_2_S-releasing hydrogels for Parkinson’s or Alzheimer’s disease remains in the exploratory stage. Cross-disciplinary approaches combining materials science and neurobiology show promise for enhancing H_2_S delivery efficiency to the central nervous system, thereby enabling novel strategies for the targeted treatment of neurodegenerative diseases.

### Cardiovascular therapy

Cardiovascular diseases remain a leading cause of death worldwide. H_2_S has demonstrated cardioprotective effects in various pathological conditions, including arrhythmia, cardiac fibrosis, heart failure, cardiac hypertrophy, ischemia–reperfusion injury, and myocardial infarction.^103^,^104^ The cardioprotective mechanisms of H_2_S include regulation of vascular tone, enhancement of endothelial function, inhibition of vascular remodeling, reduction of oxidative stress, and suppression of inflammation.^8^,^105^,^106^ Advances in hydrogel technology have enabled the exogenous delivery of H_2_S, showing promising therapeutic potential for cardiovascular disease treatment.

Atherosclerosis is a primary cause of myocardial infarction. Rupture of atherosclerotic plaque triggers platelet aggregation and thrombus formation, which can partially or completely occlude the vessel, leading to acute myocardial ischemia.^107^,^108^,^109^ Studies have shown that H_2_S-releasing hydrogels can locally deliver H_2_S after myocardial infarction, thereby attenuating ischemia–reperfusion injury and promoting cardiomyocyte survival and repair.^63^,^70^ Liang et al.^70^ developed a conductive H_2_S-releasing hydrogel loaded with adipose-derived stem cells, synthesized using 2-aminopyridine-5-thiocarboxamide, partially oxidized alginate, and tetraaniline. Upon injection into the infarcted myocardium, this hydrogel was able to restore left ventricular geometry, improve left ventricular function, increase ejection fraction, and inhibit left ventricular remodeling. Reverse transcription-polymerase chain reaction analysis further showed that the oxidized alginate/alginate–tetraaniline–2-aminopyridine-5-thiocarboxamide/gelatin/adipose-derived stem cells hydrogel significantly downregulated the expression of the inflammatory cytokine tumor necrosis factor-alpha and the pro-angiogenic factors vascular endothelial growth factor A and angiopoietin-1.^70^ A similar outcome was observed in another study involving a Pluronic F-127/KAT hydrogel incorporating 4-aminobenzothioamide as the H_2_S donor. Injection of the hydrogel onto the surface of the infarcted heart led to a reduction in infarct size and microvascular obstruction, accompanied by decreased expression of the inflammatory cytokines interleukin-1β, interleukin-6, and tumor necrosis factor-alpha.^63^ Additionally, this study also showed that hydrogel-released H_2_S significantly promoted NO production in human umbilical vein endothelial cells, indicating a potential synergistic cardioprotective effect of H_2_S and NO.

Atherosclerotic stenosis is a common complication following vascular interventional procedures, such as coronary and peripheral angioplasty, and significantly impairs therapeutic outcomes and patient prognosis. A major underlying mechanism involves incompetent endothelium, excessive proliferation of vascular smooth muscle cells and intimal hyperplasia.^110^ Therefore, maintaining the functional balance between vascular smooth muscle cells and endothelial cells is essential for reducing the risk of atherosclerotic stenosis. *in vitro* studies have demonstrated that incorporating a H_2_S donor can inhibit the proliferation and migration of smooth muscle cells, while promoting the proliferation and migration of endothelial cells.^28^,^46^,^111^,^112^ One study based on a self-assembling peptide H_2_S-releasing hydrogel further demonstrated that this system could effectively limit intimal hyperplasia in human saphenous vein segments harvested from patients undergoing bypass surgery.^46^ This was the first demonstration of such an effect in an *ex vivo* human vein culture model.^46^ Another study developed an intelligent hydrogel coating (C-HA-Cys-Allicin) composed of catechol-modified HA and cystamine, loaded with the H_2_S donor allicin. The presence of allicin in the coating reduced platelet adhesion, fibrinogen denaturation, and thrombosis formation. *in vivo* implantation of the C-HA-Cys-Allicin coating into the abdominal aorta of Sprague-Dawley rats inhibited new tissue hyperplasia and the aggregation of synthetic smooth muscle cells, demonstrating the hydrogel’s excellent biocompatibility.^28^

### Regulation of vascular tone and angiogenesis

Similar to NO, H_2_S was first recognized as an endogenous vasodilator.^103^ However, subsequent studies revealed that the vascular regulatory effects of H_2_S are bidirectional, encompassing both vasodilation and vasoconstriction.^7^,^113^,^114^ The underlying mechanisms include modulation of potassium channel activity, activation of endothelial NO synthase, and increased cyclic guanosine monophosphate levels in vascular smooth muscle cells.^113^,^114^,^115^ H_2_S also regulates angiogenesis, the formation of new blood vessels from pre-existing vasculature.^115^,^116^ Studies have shown that H_2_S promotes *in vitro* angiogenic responses by enhancing endothelial cell proliferation and migration, potentially via activation of the phosphatidylinositol 3-kinase/protein kinase B and mitogen-activated protein kinase/extracellular signal-regulated kinase signaling pathways and upregulation of vascular endothelial growth factor expression.^44^,^116^,^117^,^118^ The role of H_2_S in maintaining vascular homeostasis and function underscores its therapeutic potential in hypertension, diabetic vasculopathy, and chronic wound healing.^119^

*In vitro* studies have demonstrated the angiogenesis potential of H_2_S-releasing hydrogels. Human umbilical vein endothelial cells treated with H_2_S-releasing hydrogels showed enhanced capillary maturity, along with a greater number of branching points and longer branch lengths compared with controls.^120^,^121^,^122^,^123^ The angiogenic potential of H_2_S-releasing hydrogels has been demonstrated in the mouse model of acute hindlimb ischemia. Immunohistochemical staining for platelet endothelial cell adhesion molecule-1 (CD31) and α-smooth muscle actin revealed a significant increase in capillary and small arteriole density in mice treated with the hydrogel.^120^ Similar results were observed in rat ischemic wound revascularization models. CD31 immunohistochemistry demonstrated that, compared to controls, rats treated with GYY4137 hydrogel exhibited increased perfusion in the distal skin flap at day 14, along with a more extensive vascular network within the healing wound.^121^

### Regulation of bone metabolism

H_2_S plays multiple roles in bone metabolism and bone-related diseases, including regulation of bone marrow stem cells and bone formation, modulation of osteoblast and osteoclast activity, maintenance of bone homeostasis, and its anti-inflammatory and antioxidant effects.^7^ H_2_S donors have shown significant effects in the treatment of osteoporosis, osteoarthritis, fracture healing, and periodontal diseases, and are considered potential therapeutic agents for bone disorders in the future.^124^

*In vitro*, incorporating H_2_S donors effectively promoted the differentiation of bone marrow mesenchymal stem cells, modulated macrophages, scavenged ROS and inhibited inflammatory response and apoptosis.^125^,^126^,^127^,^128^ Bone marrow mesenchymal stem cells regulate the rate and extent of new bone formation. A co-culture study of bone marrow mesenchymal stem cells with H_2_S donors demonstrated that H_2_S treatment restored osteogenic gene expression and enhanced both alkaline phosphatase activity and calcium deposition.^126^ The imbalance between M1 and M2 macrophages plays a critical role in the onset and progression of bone metabolic diseases, as M1 polarization promotes osteoclastogenesis while M2 polarization supports osteogenesis.^129^ Macrophage senescence is also a key factor impairing fracture healing in diabetic patients, in addition to macrophage polarization. One recent study demonstrated that applying a H_2_S–releasing hydrogel to high glucose–treated macrophages downregulated senescence-related genes and effectively reversed macrophage aging.^127^

The therapeutic potential of H_2_S–releasing hydrogels has also been confirmed *in vivo*. Tao et al.^125^ developed an injectable hydrogel microsphere (GelMA@FPLBD) containing H_2_S-donor nanomicelles designed to target macrophages, which was subsequently tested in an osteoarthritis model. By encapsulating nanomicelles, the microsphere effectively maintains controlled release in rats and markedly attenuates cartilage degeneration and joint destruction in osteoarthritis. Jiang et al.^126^ investigated the protective effects of H_2_S–releasing hydrogels in osteoporosis, through developing an injectable hydrogel incorporating the S-aroylthiooxime donor. In the rat model of osteoporosis, treatment with the hydrogel significantly accelerated defect repair, with the defect area nearly completely healed within 8 weeks post-surgery. Similar therapeutic effects were reported when H_2_S–releasing hydrogels were applied to intervertebral disc degeneration. Zheng et al.^60^ encapsulated JK1 into a collagen hydrogel to develop a pH- and enzyme-responsive H_2_S release system (Col-JK1). Col-JK1 inhibited apoptosis of nucleus pulposus cells, prevented extracellular matrix degradation in intervertebral discs, and promoted repair of annular puncture-induced intervertebral disc degeneration in rat models.

Systemic diseases such as diabetes and rheumatic disorders also affect bone metabolism and regeneration. In two diabetes-related bone injury model studies, H_2_S-releasing hydrogels were applied and demonstrated therapeutic efficacy. Fang et al.^72^ developed a polyphenol-mediated alginic acid/gelatin hydrogel and applied it to a diabetic periodontitis model. This hydrogel encapsulated bovine serum albumin nanoparticles containing NaHS donors and a conductive poly(3,4-ethylenedioxythiopene)-assembled polydopamine-mediated silk microfiber network, which confers high conductivity to the hydrogel and transmits endogenous bioelectricity. This hydrogel suppressed oxidative stress and osteoclast activity while promoting osteogenesis and angiogenesis *in vivo* through coupling endogenous bioelectricity and H_2_S signals. In another study, Zhang et al.^127^ developed an injectable H_2_S delivery system (HydroWrap), in which H_2_S donors were covalently linked to the hydrogel matrix. Under near-infrared irradiation, the hydrogel network underwent contraction, thereby accelerating H_2_S release. The most pronounced bone remodeling occurred in diabetic mouse fracture models treated with HydroWrap in combination with near-infrared irradiation. Furthermore, RNA-seq analysis of RAW264.7 cells exposed to control or hydrogel treatment demonstrated that the protective effects were associated with nuclear factor kappa-B signaling, cellular senescence, and mitophagy. In addition, Geng et al.^128^ developed a dual-gas-mediated injectable hydrogel (DNRS) capable of simultaneously consuming NO and releasing H_2_S in the study of rheumatoid arthritis models. DNRS synergized with methotrexate (MTX) to exert anti-inflammatory and bone-protective effects *in vivo*, and the MTX/DNRS gel group alleviated rheumatoid arthritis symptoms and delayed disease progression.

### Wound healing

Wound healing is a complex physiological process that involves multiple cell types and molecular events, typically progressing through four overlapping stages: hemostasis, inflammation, proliferation, and remodeling.^130^ Following injury, the body initiates hemostatic mechanisms in which vasoconstriction and platelet aggregation temporarily seal the wound. Activated platelets release clotting factors that attract fibrin and other components, forming a blood clot to further close the wound. Once the clot forms, the process transitions to the inflammatory phase, which primarily removes debris and necrotic tissue, thereby laying the foundation for subsequent repair. The proliferative phase, usually beginning several days after injury, involves cell proliferation and extracellular matrix synthesis to fill the wound gap, leading to granulation tissue formation and epithelial coverage. After substantial healing, the tissue enters the remodeling phase, during which collagen is extensively replaced and reorganized, granulation tissue gradually transforms into scar tissue, and the scar’s tensile strength approaches, but rarely matches, that of normal tissue. Elasticity and function gradually improve during this stage, which may last from weeks to months, or even years.^130^,^131^ In acute or mild trauma, this sequence proceeds in an orderly manner, ultimately restoring tissue integrity. However, under pathological conditions such as diabetes or venous insufficiency, wounds often remain in a persistent inflammatory state, resulting in chronic, non-healing ulcers. As a gaseous signaling molecule, H_2_S has shown remarkable potential in wound healing due to its anti-inflammatory, pro-angiogenic, and tissue-reparative properties. Combined with the biocompatibility and moisture-retaining capacity of hydrogels, H_2_S-releasing hydrogels have emerged as a research focus for treating chronic wounds, burns, diabetic ulcers, and infected injuries.^59^

H_2_S-releasing hydrogels have demonstrated promising therapeutic potential in acute wound healing. Evidence from multiple animal wound models indicates that these hydrogels facilitate hemostasis, enhance fibroblast migration, and promote wound epithelialization.^123^,^132^ Treatment with H_2_S-releasing hydrogels during inflammatory phase suppresses inflammatory responses, attenuates oxidative stress, and reduces neutrophil infiltration, thereby improving wound healing quality through modulation of the wound microenvironment.^44^,^71^,^123^ Notably, macrophages are key inflammatory cells in wound repair. *in vitro* and *in vivo* studies reveal that incorporating H_2_S donors induces macrophage polarization toward the M2 phenotype, thereby alleviating wound inflammation and enhancing tissue remodeling.^44^,^61^,^133^ Furthermore, during the proliferative and remodeling phases, H_2_S-releasing hydrogels promote angiogenesis and collagen deposition, thereby accelerating wound closure.^123^,^132^,^133^,^134^ However, studies showed that H_2_S concentration is a critical determinant of wound healing outcomes, with one such study reporting that wounds treated with an alginate/H_2_S 0.5% hydrogel exhibited a higher closure rate compared with those treated with 5% or 10% H_2_S, along with the highest scores for epithelialization and collagen synthesis.^132^

Additionally, H_2_S-releasing hydrogels facilitate infected wound healing through multiple mechanisms, including antibacterial, anti-inflammatory, and tissue-regenerative effects. Studies have suggested that endogenous bacterial H_2_S protects bacteria from antibiotic-induced damage through its antioxidant activity.^135^,^136^ However, subsequent research revealed that the antibacterial mechanism of H_2_S is multifaceted and strain-specific. The antibacterial efficacy of exogenous H_2_S depends on whether bacteria can synthesize endogenous H_2_S.^137^ For strains producing little or no endogenous H_2_S (such as *Acinetobacter baumannii*), exogenous H_2_S can disrupt membrane permeability and interfere with metabolism, thereby directly enhancing antibiotic bactericidal effects.^138^ Several studies have demonstrated that integrating H_2_S donors into hydrogels effectively exerts antibacterial effects in both *in vitro* and *in vivo* settings. Qian et al.^45^ assessed the antibacterial activity of an H_2_S-releasing dipeptide hydrogel (SATO-phenylalanine-glutamic) and reported that, compared with a non–H_2_S-releasing control hydrogel, SATO-phenylalanine-glutamic achieved a 97% inhibition rate against planktonic *Staphylococcus aureus*, more effectively limited biofilm formation, and disrupted pre-formed biofilms. Yang et al.^71^ developed a ROS-responsive H_2_S-releasing hydrogel (PTCM@PMet NPs/F127-P) utilizing PeroxyTCM as the donor, and revealed that, relative to the control group, the H_2_O_2_/PTCM@PMet NPs group exhibited higher inhibition rates against *Escherichia coli* and *Staphylococcus aureus*. Through incorporating antimicrobial agents, H_2_S-releasing hydrogels synergistically promote the healing of infected wounds. For example, hydrogels encapsulated with keratin-based H_2_S donor, ciprofloxacin, and anthocyanins previously developed by Liu et al.^64^ can inhibit the growth of *Escherichia coli* and *Staphylococcus aureus in vitro*. Carboxymethyl chitosan is a biocompatible biopolymer with notable antibacterial activity, owing to its ability to disrupt bacterial membranes. One recent study incorporated the H_2_S donor (N-(benzoyl mercapto) benzamide) into HA-carboxymethyl chitosan hydrogels to create a hydrogel-based bioactive synthetic skin. Hydrogel-based bioactive synthetic skin exhibited potent antibacterial activity against *Pseudomonas aeruginosa*, *Escherichia coli*, and *Staphylococcus aureus*, accelerated the healing of infected wounds in animals, and lowered the risk of scar formation.^133^ E-Polylysine, a cationic surfactant with broad-spectrum antibacterial activity, has also been used to modify H_2_S-releasing hydrogels. Colony formation inhibition assay of *Escherichia coli* and *Staphylococcus aureus* confirmed the excellent antimicrobial properties of the E-polylysine-modified hydrogels.^44^,^139^ In addition to aforementioned drugs, other compounds, such as bis[tetrakis(hydroxymethyl)phosphonium] sulfate, phenylboronic acid–functionalized silver nanoparticles, and sodium copper chlorophyllin, have also been incorporated into H_2_S-releasing hydrogels, further enhancing their antibacterial potency and accelerating the repair of infected wounds.^140^,^141^,^142^ These findings highlight the therapeutic potential of H_2_S-releasing hydrogels for infected wound treatment.

Chronic wounds, such as diabetic ulcers, are challenging to heal due to their complex pathogenesis, causing significant physical suffering and economic burden for patients. Prolonged hyperglycemia often causes vascular pathology, reducing blood supply to the wound and impairing cellular proliferation and tissue repair. Hyperglycemia also disrupts inflammatory regulation and increases oxidative stress, creating a persistent inflammatory state that further impairs wound healing.^143^ Numerous studies have demonstrated the efficacy of H_2_S-releasing hydrogels in promoting the healing of diabetic wounds. Compared with controls or hydrogels without H_2_S, H_2_S-releasing hydrogels alleviate wound inflammation, reduce oxidative stress, and promote fibroblast proliferation, collagen deposition, and angiogenesis.^64^,^122^,^140^,^144^,^145^ Infection is also a frequent complication of diabetic wounds. Impaired immunity and poor wound perfusion facilitate the spread of infections from superficial to deeper tissues, and uncontrolled infection or ischemia may even lead to amputation. Therefore, effective infection management is crucial for the treatment of diabetic wounds. As previously mentioned, studies highlight that H_2_S-releasing hydrogels enhance antimicrobial defense in diabetic wounds.^122^,^140^ In summary, H_2_S-releasing hydrogels provide a novel therapeutic approach for managing diabetic wounds, potentially enhancing impaired healing and reducing the risk of severe complications, including amputation.

### Other applications of H_2_S-releasing hydrogels

In addition to the aforementioned applications, H_2_S-releasing hydrogels have also been investigated in tumor therapy, tissue regeneration, and metabolic regulation. Increasing evidence suggests that H_2_S exerts a bidirectional regulatory effect on tumor development: endogenous or low concentrations of exogenous H_2_S are thought to promote tumor development, while high doses of exogenous H_2_S suppress tumor proliferation.^146^,^147^ Given this property, various hydrogels capable of modulating endogenous or exogenous H_2_S levels have been designed and evaluated in tumor models. Qiu et al.^148^ developed a chondroitin sulfate hydrogel loaded with sulfate-reducing bacteria (SRB@ChS Gel), in which the bacteria metabolized chondroitin sulfate to continuously produce H_2_S. SRB@ChS Gel exhibited significant cytotoxicity against tumor cells *in vitro* by disrupting mitochondrial cytochrome C oxidase. Breast cancer model mice treated with SRB@ChS Gel showed enhanced activation of dendritic cells and CD8^+^ T cells, accompanied by suppressed tumor growth, recurrence, and distant metastasis. The anti-tumor effect of H_2_S-releasing hydrogels has also been shown in glioma models.^149^ A porphyrin-based metal-organic framework was incorporated into a dual-network cross-linked HA hydrogel nanocomposite to inhibit glioma cell proliferation. ROS and H_2_S were generated from the hydrogel in response to the elevated glutathione levels in the tumor microenvironment, combining gas therapy with sonodynamic therapy to synergistically enhance the killing effect on tumors.^149^

H_2_S-releasing hydrogels demonstrate significant potential in stem cell therapy by integrating the biological functions of the gasotransmitter H_2_S with the controlled delivery capabilities of hydrogels. In recent years, accumulating studies have focused on the effects of H_2_S-releasing hydrogels in regulating stem cell differentiation and tissue repair. In one study, periodontal ligament stem cells cultured on H_2_S-releasing hydrogels showed higher expression levels of osteogenic markers osteocalcin and alkaline phosphatase when the hydrogels contained BNP (bovine serum albumin nanoparticles encapsulating NaHS) compared with periodontal ligament stem cells on BNP-free hydrogels, indicating that H_2_S promotes osteogenic differentiation.^72^ Similar effects were observed in bone marrow mesenchymal stem cells, where the incorporation of H_2_S donor-loaded hydrogels enhanced ALP activity.^126^ Another study reported that induced neural stem cells loaded on anisotropic ferrofluid hydrogels, constructed from ferric tetrasulfide, carboxymethyl chitosan, and gold, differentiated into neurons with longer, directionally aligned axons, thereby facilitating neural regeneration and functional recovery following SCI.^83^ The ischemic and hypoxic microenvironment often induces stem cell apoptosis during transplantation, while H_2_S-releasing hydrogels significantly enhance stem cell survival by scavenging ROS and suppressing the mitochondrial apoptosis pathway. In a rat model of SCI, hydrogels composed of dental pulp stem cells and mesoporous silica loaded with the H_2_S donor JK were shown to downregulate the pro-inflammatory cytokines interleukin-6 and tumor necrosis factor-alpha, attenuate microglial pyroptosis, and promote the proliferation and differentiation of dental pulp stem cells.^82^ Similarly, the conductive H_2_S-releasing hydrogel developed by Liang et al.^70^ prolonged the retention of adipose-derived stem cells in myocardial infarction models by encapsulating adipose-derived stem cells and continuously releasing H_2_S.

In addition, H_2_S-releasing hydrogels influence metabolic regulation *in vivo* by enabling the targeted delivery of H_2_S. In a study utilizing SATO-based hydrogels, controlled doses of H_2_S were delivered to subcutaneous adipose tissue in mice.^150^ It was demonstrated that both low and high doses of H_2_S significantly increased fat accumulation in wild-type mice, whereas CSE knockout mice exhibited increased fat accumulation only under high-dose conditions. The results suggested that adipose tissue was more sensitive to exogenous H_2_S in the presence of endogenous H_2_S synthesized by CSE, highlighting the essential role of endogenous H_2_S in the regulation of fat metabolism.^150^

## Clinical Translation Challenges of Hydrogen Sulfide-Releasing Hydrogels

Although H_2_S-releasing hydrogels have shown promising results in various biomedical applications, the translation from preclinical research to clinical application remains highly challenging (**Figure 7**). As previously discussed, biological effects of H_2_S are strongly concentration-dependent, resulting in a narrow therapeutic window.^151^ Physiological levels of H_2_S promote anti-inflammatory and pro-angiogenic effects, whereas excessive levels may induce severe adverse outcomes, including neurotoxicity and respiratory suppression.^7^ Several micromolecular H_2_S donors also display uncontrolled release kinetics or are sensitive to fluctuations in the physiological microenvironment (e.g., pH, ROS, or enzymatic activity), which may cause burst release or insufficient sustained delivery, thereby complicating reproducible *in vivo* exposure–response relationships.^152^,^153^ Furthermore, H_2_S donors vary substantially in their release mechanisms, byproduct profiles, and metabolic pathways. The long-term biosafety, accumulation potential, and possible immunogenicity of these byproducts have yet to be systematically assessed in clinical settings.^154^ Standardized investigations into biodegradation pathways, metabolic product characterization and comprehensive toxicological assessments are urgently required. In addition, the lack of reliable and standardized methods for *in vivo* H_2_S pharmacokinetic measurement, as well as the absence of quantifiable biomarkers correlated with therapeutic efficacy, presents significant challenges for early dose optimization and efficacy prediction. Current techniques commonly used to evaluate H_2_S release kinetics include the methylene blue assay, H_2_S-selective electrochemical probes, and H_2_S-selective fluorescent probes. However, the kinetic data obtained from these methods are generally suitable only for comparative analyses among donors under identical experimental conditions and may fail to accurately represent the actual H_2_S release behavior in physiological environments.^152^

**Figure 7:**
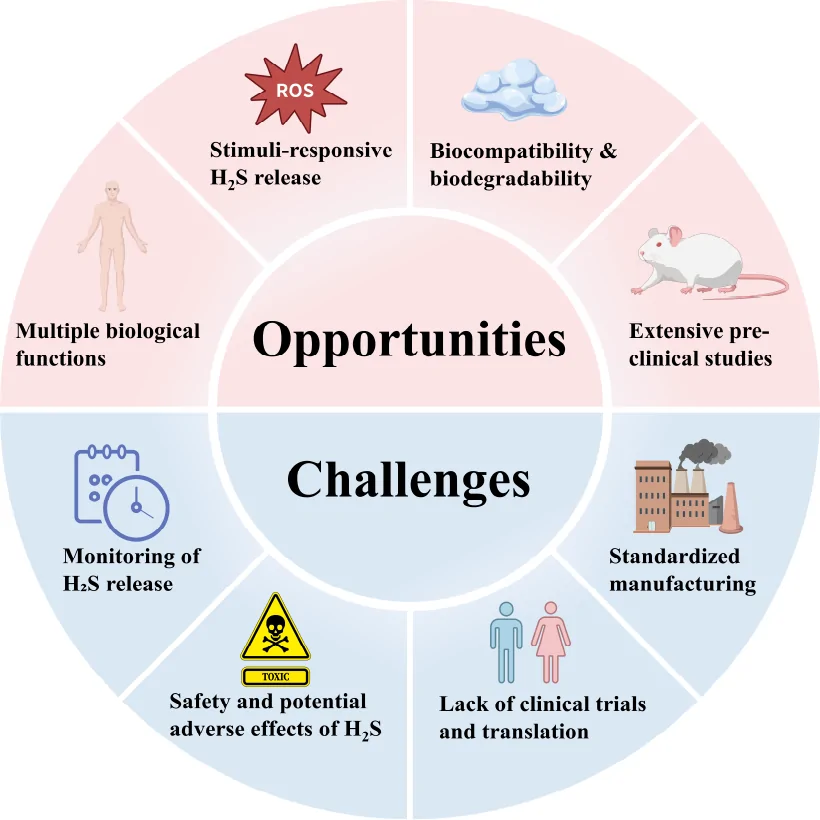
Opportunities and challenges of H_2_S-releasing hydrogels. H_2_S-releasing hydrogels offer promising opportunities due to multiple biological functions, the stimuli-responsive release behavior, good biocompatibility and biodegradability, and extensive pre-clinical studies. Challenges remain in precise monitoring of H_2_S release, safety and potential toxicity, limited clinical evidence, and standardized large-scale manufacturing. Created with PowerPoint 16.101.3 and BioGDP.com. H_2_S: Hydrogen sulfide

The biocompatibility, degradability, immune response and sterilization stability of H_2_S-releasing hydrogel must also be considered in clinical translation.^155^,^156^ Currently, most studies assessing the biocompatibility of H_2_S-releasing hydrogels remain at the cellular level, with limited long-term *in vivo* data. Extrapolating findings from *in vitro* or animal models to humans presents challenges, highlighting the necessity for comprehensive safety evaluations to prevent potential adverse reactions in patients.^157^ Moreover, the degradation rate of hydrogels should ideally match the tissue regeneration process; however, achieving precise control over degradation kinetics both *in vitro* and *in vivo* remains a major obstacle.^157^ Another key challenge in clinical translation involves the risk of off-target effects, which may cause unintended H_2_S release in non-target tissues and lead to systemic side effects. For instance, in one study on gelatin methacrylate hydrogels loaded with NaHS or GYY4137, it remains unclear whether locally or regionally delivered H_2_S donors undergo systemic diffusion, warranting further investigation.^121^ The transition from laboratory-scale preparation to clinical-grade large-scale production presents additional challenges. Ensuring the consistency of hydrogel physicochemical properties, such as pore size, crosslinking density, swelling ratio, and mechanical strength, as well as maintaining consistent H_2_S release efficiency, remains difficult. Variations in these parameters substantially affect manufacturing reproducibility and quality control, thereby influencing clinical reliability and hindering regulatory approval of H_2_S-releasing hydrogel products.

In summary, the development of next-generation H_2_S delivery systems with intelligent and precisely controllable release characteristics is urgently required. Furthermore, the establishment of representative large-animal models and the execution of well-designed clinical trials are also essential to better predict human therapeutic outcomes. The clinical translation of H_2_S-releasing hydrogels remains a long and challenging endeavor, and achieving these goals will require close interdisciplinary collaboration among chemists, materials scientists, pharmacologists, and clinicians.

## Limitations

Although this review comprehensively summarizes biomedical applications and translational prospects of H_2_S-releasing hydrogels, it also has several inherent limitations. First, the literature search was confined to English-language publications, raising the possibility that pertinent studies indexed elsewhere or published in other languages were not captured. Second, considerable heterogeneity exists across the included studies in terms of hydrogel composition, H_2_S donors, release kinetics assessment, and experimental models, which limits direct comparability and may introduce interpretative bias. Third, the current evidence base is predominantly derived from *in vitro* experiments and small-animal models, with a paucity of large-animal or clinical data, which limits the translational applicability of the findings.

## Conclusion

H_2_S-releasing hydrogels have attracted considerable attention as an emerging biomaterial strategy, owing to their integration of the physiological functions of H_2_S with the properties of hydrogels. A wide spectrum of H_2_S donors, including inorganic sulfides, GYY4137, SATO and JK donors, have been incorporated into both natural and synthetic hydrogel matrices. Through chemical or physical crosslinking, rational donor selection and incorporation strategy, their key properties, such as mechanical strength, stimulus responsiveness, and release kinetics, can be precisely tuned to construct delivery systems with tailored functionalities. H_2_S-releasing hydrogels have demonstrated broad applications across multiple biomedical fields. In neurological disorders, H_2_S-releasing hydrogels effectively suppress inflammation and oxidative stress, protect neuronal cells, and promote functional recovery. In cardiovascular disorders, H_2_S-releasing hydrogels enhance endothelial cell growth while inhibiting smooth muscle cell proliferation, maintain vascular homeostasis and improve cardiac function. In bone-related disorders, H_2_S-releasing hydrogels regulate osteoblast and osteoclast activity to maintain bone homeostasis. Wound healing hydrogels that release H_2_S exert anti-inflammatory, pro-granulation, and antimicrobial effects, thereby accelerating tissue repair and reducing the risk of complications. Furthermore, H_2_S-releasing hydrogels have also shown therapeutic potential in cancer treatment and stem cell regulation. Despite the significant promise demonstrated in preclinical studies, further efforts are required to strengthen clinical translation and advance these systems toward safe and effective therapeutic applications.

**Table 1:** The release kinetics differences among various types of H_2_S donors within hydrogels

H_2_S donors	Material	Incorporation strategy	Release kinetics	Reference
NaHS	Silk fibroin hydrogel	Physical encapsulation Physical encapsulation	Moderate release, peaking at 2–4 h, followed by sustained release detectable over 24 h	80 89
	bovine serum albumin nanoparticle-loaded algin/gelatin hydrogel	Nanoparticle encapsulating	Slow and sustained release, peaking at 21 d	72
	Gelatin methacrylate (GelMA)	Physical encapsulation	Moderate release, sharply peaking at 8 h, followed by rapid decrease	121
GYY4137	Gelatin methacrylate (GelMA)	Physical encapsulation	Slow and steady release for 14 d	121
	thiol-modified carboxylated nanocellulose and acrylic acid	Physical encapsulation	pH-dependent release, rapid at low pH (6.0), slower at high pH (7.4), peaking at 2–4 h	139
JK1	Collagen hydrogel	Physical encapsulation	pH-responsive release, burst releasing at pH 6.0 (peak time ~90 to 200 min) and gradual decline; slow releasing at pH 7.4 (peak time ~ several to tens of hours)	60
	Hyaluronic acid hydrogel	Physical encapsulation		61
	Sodium alginate hydrogel	Physical encapsulation		62
JK2	Mesoporous silica nanoparticle-loaded Pluronic F127 hydrogel	Nanoparticle encapsulating	pH-dependent release, burst releasing within first few hours at pH 6.4, gradual decline within 60 h; near zero-order release at pH 7.4 for 24 h, then stable	82
Allicin	Catechol hyaluronic acid/Cys hydrogel	Physical encapsulation	Moderate release, peaking at 12 h	28
DATS	Mesoporous silica nanoparticle-loaded PF127/hydroxymethyl cellulose/sodium alginate-derived hydrogel	Nanoparticle encapsulating	Slow release, peaking over several days	120
STAO	Peptide hydrogel	Covalent bonding	Cysteine-activated release, variable rate with tunable peak from minutes, hours to tens of hours via peptide design (e.g., peptide sequence, substituents, linker)	45 46 65 66 67 68 150
	Sodium alginate/PEGDA double-network gel	Covalent bonding	Cysteine-activated sustained release, slow releasing for 7 d	126
SATO modified ε-PL	Carboxymethyl chitosan/oxidized hyaluronic acid hydrogel	Physical encapsulation	Moderate release, peaking at 12 h	44
4-Aminobenzothioamide coupled keratin	Pluronic F-127 hydrogel	Physical encapsulation	Moderate release, peaking within several hours, then plateau	63
Thiobenzoic acid coupled keratin	Poly(γ-glutamic acid) hydrogel	Physical encapsulation	Moderate release, burst releasing within first 500 min, followed by sustained release for 52 h	64
Peroxythiocarbamate (PeroxyTCM)	mPEG-PMet nanoparticle-loaded F127-poly(l-aspartic acid-N-hydroxysuccinimide) hydrogel	Nanoparticle encapsulating	ROS-responsive release, slow releasing under low ROS, generally sustained for tens of hours	71
	mPEG-PMet nanoparticle-loaded methoxy poly(ethylene glycol)-polyalanine-polyphenylalanine hydrogel	Nanoparticle encapsulating		49
	Zn-CA nanoparticle-loaded lipoic acid modified chitosan/gelatin methacryloyl hydrogel	Nanoparticle encapsulating		48
2-Aminopyridine-5-thiocarboxamide	Oxidized alginate and gelatin	Covalent bonding	Fast release, peaking at 30 min, then stable	70
N-(benzoyl mercapto) benzamide	Hyaluronic acid-carboxymethyl chitosan hydrogel	Physical encapsulation	Slow release, peaking over several days	133

**Facts**

1. Incorporating hydrogen sulfide (H_2_S) donors into hydrogels enables localized, sustained, and controllable H_2_S delivery, overcoming the instability and systemic toxicity limitations of free H_2_S.
2. Advances in donor chemistry and hydrogel engineering improve the control over release kinetics, biocompatibility, and tissue-specific targeting.
3. Studies have demonstrated the therapeutic potential of H_2_S-releasing hydrogels in neuroprotection, cardiovascular therapy, ischemia-reperfusion injury, wound healing, and tissue regeneration.

**Open Questions**

1. The optimal H_2_S dosing and release profiles required to balance therapeutic efficacy and safety across different disease contexts remain to be definitively established.
2. Standardized and physiologically relevant methods for quantifying H_2_S release from hydrogels are still lacking.
3. Appropriate large-animal models that can reliably predict human responses to H_2_S-releasing hydrogels need to be established.
4. Regulatory and quality-control frameworks for advancing H_2_S-releasing hydrogels toward clinical trials remain insufficiently developed.

## Data Availability

*Not applicable.*
